# Supplementary material for: Optimal spectral templates for triggered feedback experiments
Source: PLoS One. 2020 Apr 28;15(4):e0228512. doi: 10.1371/journal.pone.0228512 (PMC7188275; doi:10.1371/journal.pone.0228512)
Supplement: S1 Fig — (PDF) [file pone.0228512.s001.pdf]

A simple 2-d example

To illustrate the action of our algorithm, we constructed a simple two-dimensional example using computer-generated data. We selected 50 points each from three Gaussian distributions. The 50 points from one Gaussian (green circles, Fig S1A) were considered targets and the other 100 points were distractors (magenta circles, Fig S1A). The average template (green filled circle, Fig S1A) is the centroid of the target data points. The initial threshold (broken-line circle, Fig S1A) was determined by smoothing the distance distributions as above and calculating their crossing point.

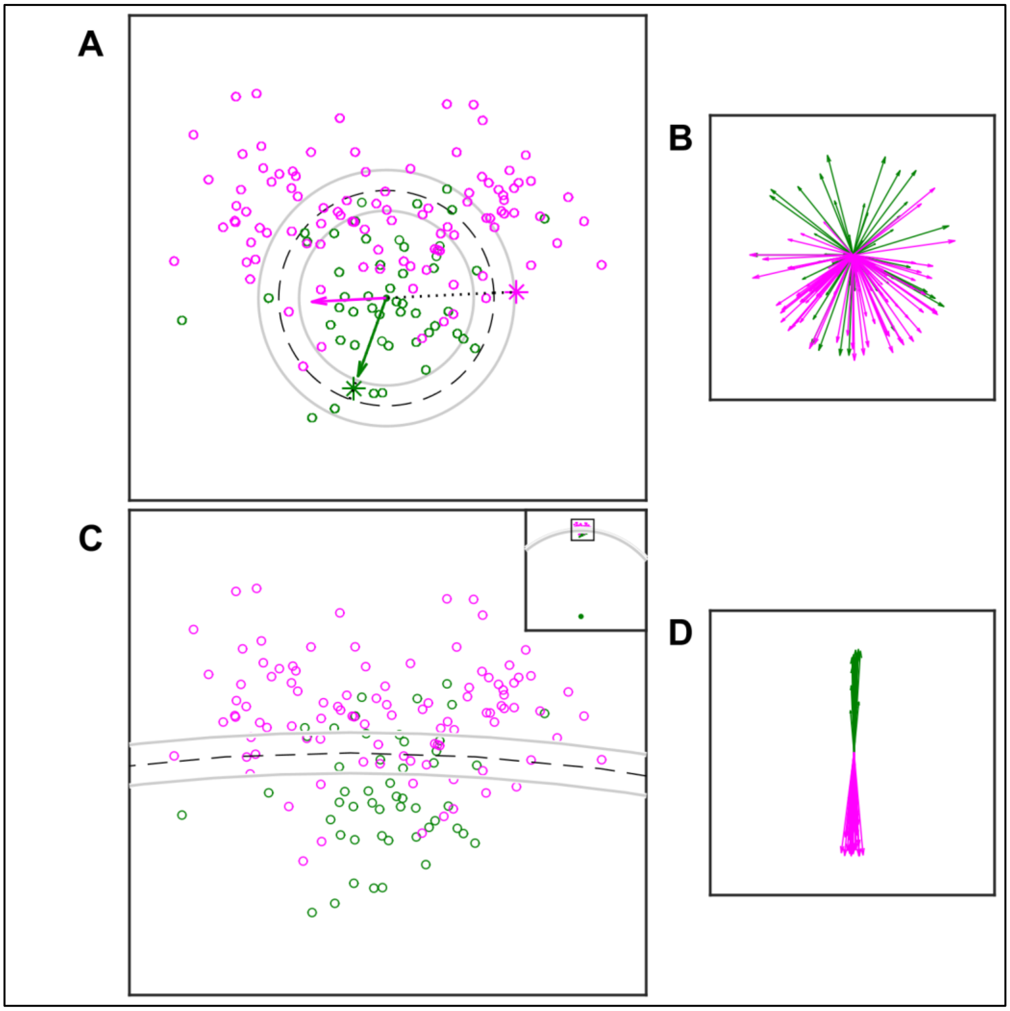

Fig S1. **Illustration of the gradient expression** (A) Target (green circles) and distractor (magenta circles) points from computer-generated data. The centroid (filled green circle) of the target points is the initial averaged template. The threshold distance is indicated by the dotted circle and +/- 0.5 times the standard deviation of the smoothing Gaussian are indicated in gray. Asterisks show example target and distractor points; arrows show the corresponding contribution to the gradient. (B) The contribution to the gradient from all target and distractor slices (for the template in A). Green vectors are roughly circularly symmetric, whereas magenta vectors have a net downward orientation. (C) Same as A but with the threshold (dotted and gray lines) corresponding to the optimized template. The inset shows that the optimized template (filled green circle) is at some distance from all points, leading to a more linear decision threshold. The area marked by the square in the inset is shown magnified in the main panel (D) The contribution to the gradient from all target and distractor slices for the template in C. The green and magenta vectors are balanced, leading to net zero gradient vector.

The magnitude of the contribution to the negative gradient from a single slice, as mentioned in the derivation, is high for points near the threshold boundary (magenta and green asterisks, Fig S1A). The target points that are at a near-threshold distance from the template are scattered around the decision surface, but similar distractor points are all on one side. Thus, the contributions to the negative gradient from the distractor points act to move the template downward (Fig S1B). As we step through gradient descent, this process continues until the template vector moves a substantial distance downward (Fig S1C, inset). At this location both the target and distractor slices are located above the template, with the gradient vectors for the target and distractor points cancelling in a push-pull manner (Fig S1D). Note that moving the

template far away from the data points, along with a much greater distance threshold, has the effect of making the decision surface approximate a linear boundary between the two distributions (Fig S1C).
