# Supplementary material for: Optimal spectral templates for triggered feedback experiments
Source: PLoS One. 2020 Apr 28;15(4):e0228512. doi: 10.1371/journal.pone.0228512 (PMC7188275; doi:10.1371/journal.pone.0228512)
Supplement: Attachment — Submitted filename: KulkarniTroyerResponse to reviewers.docx [file pone.0228512.s002.docx]

**Response to previous reviews**

We thank the reviewers for their comments. For each reviewer, we respond to the central critiques. All minor/specific comments have been addressed.

We decided to move the illustration of our method using simulated data to supplemental materials. This illustration may be illuminating for some readers, but it may distract others from the main results.

**Reviewer #1**:

“The authors must demonstrate that their technique can actually drive sensorimotor learning, rather than simply improve targeting on a dataset of unmanipulated song.” The reviewer refers to a PLoS ONE article (Pearre et al. 2017). However, like our paper, this paper reports targeting accuracy on a dataset of unmanipulated song. More generally, many previous studies have successfully used a spectral template to drive sensorimotor learning. The empirical question is not whether our method can drive learning, but whether the improved accuracy resulting from our method is more effective at driving learning. Given the large individual variability in birds and syllables, answering this question would require extensive experimentation across several experimental paradigms. This is beyond the scope of this paper.

“Please include an analysis that demonstrates how much training data is required.” We thank the reviewer for this suggestion and now include it. The results make it clear that our approach can reach near asymptotic accuracy with very small training sets. This is a large practical benefit, and we contrast this with previously published machine learning approaches that can be more accurate but use significantly more training data.

“Please also show how sensitivity and precision change over the course of experiments for the optimized templates.” The reviewer seems to have a particular behavioral manipulation in mind: shifting pitch of the targeted syllable. While this is the most well-known use of triggered feedback, this is but one use of the technique. We have expanded our Introduction to clarify the range of experimental designs that could benefit from our approach. Furthermore, one advantage of small training sets needed for our method is that templates can be recalculated at regular intervals throughout the experiment to track changes in syllable phonology.

“The code as presented does not currently meet the standards established for open-source software.” We thank the reviewer for this suggestion. We have implemented the following changes in our code/documentation package:

- We have now placed our code and documentation in a GitHub repository along with an open source license (https://github.com/toddtroyerlab/template_optimization).
- We have streamlined the code such that a user interested only in end-to-end analysis can do so by running one script and changing a handful of lines in that script.
- We have updated our documentation and made it more streamlined.
- An ‘aficionado’ who may want to customize the code will now find enough documentation and details in each function. The GitHub interface will also allow easier interaction between authors and users.

**Reviewer #2**:

“The work is novel and appears to be a genuine advance over prior methods. In addition, this work would be of significant interest to those working with songbirds on conditioned/triggered feedback experiments.” We thank the reviewer for positive evaluating the significance of our work.

The central critique was a “big picture confusion/ambiguity that runs throughout the manuscript. What is the aim of this template construction method – detecting temporally precise slices or identifying syllables?” This ambiguity is inherited from the field: several studies use TF to target precise slices, e.g. for shifting pitch, whereas others use TF to identify syllables, e.g. for changing sequence or timing or when disrupting learning of specific syllables. Both types of experiments could benefit from our approach.

To address the confusion, we have reworked the transition from the analysis of temporal slices to our more syllable-based analyses, shortening this transition and removing an extraneous figure (previous figure 5). We have also expanded our introduction and reference list to give a sense of the variety of ways that TF has been used in the field.

“With what reliability can you distinguish target and distractor slices from within the same syllable instance?” This question is complicated by the fact that many song syllables have portions of the syllable where the spectrum is relatively constant. By definition, different slices within these portions of song would be hard to distinguish. Addressing the reviewer’s question would require segmenting song at the subsyllabic level, something that is difficult to do in a rigorous and consistent manner. Most implementations of TF prevent multiple triggers in one syllable by disabling the trigger for a short period after a given trigger (on the order of a length of a syllable). Instead of using a fixed time period, we excluded slices from within the same syllable as counting as a match.

“If reliably detecting the position in the song ultimately requires looking for consecutive matching slices, the temporal specificity of the detection will significantly suffer.” The reviewer’s intuition is not correct. A condition that says trigger on the third match to the template may be just as precise as a condition that says trigger on the first match to the template. We now report temporal jitter and show that for accurately targetable syllables, targeting is temporally precise.

“Slices from inter-syllable gaps were tossed from further analysis.” The easiest way to prevent corruption from gaps is to require a spectral slice to exceed a simple amplitude threshold and to be close to the template to be considered a template match. For each bird, we determined a fixed threshold by examining the amplitude distribution for gaps and syllables in our training set and have redone our analysis by including suprathreshold gaps.

“Gradient decent was run until termination conditions are met.” We now report the statistics of termination of our gradient descent algorithm.
