# Supplementary material for: Optimal spectral templates for triggered feedback experiments
Source: PLoS One. 2020 Apr 28;15(4):e0228512. doi: 10.1371/journal.pone.0228512 (PMC7188275; doi:10.1371/journal.pone.0228512)
Supplement: Attachment — Submitted filename: Response_to_reviewersRev.docx [file pone.0228512.s003.docx]

Response to previous reviews.

We thank the reviewers for their positive comments on our manuscript. Here we address the specific concerns raised by reviewer #1.

*1. The organization of Results section appears to be confusing. The first subsection of Results “Template optimization using gradient descent” appears to be a mixture of methods and results.*

It is often uncertain how to divide models or theoretical analysis into “methods” and “results.” We have reorganized the manuscript so that the derivation of our gradient algorithm is included in the Methods section. This section includes some example data to illustrate the method, but all the analysis of performance and group data is presented in Results. We have re-edited the beginning of the Results section to smooth the transition and present our overall approach.

*2. In “Calculating averaged templates” in Methods section, the authors explained how the second slice was calculated as a weighted sum of the first (1/15) and the second slice (14/15). Should the second slice be 2/15 or 14/15?*

We have tried several iterations of detailing the calculations that underlie alignment by “linear stretching of time.” At this point we believe that the intuition of linear stretching is sufficiently clear, and have dropped the sentences attempting to detail the math.

*3. In the caption of Fig 1., “and” is redundant in the sentence: For visual clarity, inter-syllable gaps and are not shown.*

Done. *4. The authors may identify more literature that is similar to [3] and include those in the citation in the sentence:*

*Our work is based on a popular implementation of TF…birdsong field, to demonstrate this specific field is actually popular and thus enhance the significance of this work.* The references describing the use of triggered feedback in the songbird field were presented in the previous paragraph. We now re-reference the articles that use an averaging approach to constructing templates (most of the references). We retain the single reference [3] where we talk about exactly mimicking the real-time algorithm known as EvTAF, first published in [3].

*5. Instead of stretching the shorter instances and compressing longer instances to match the majority - those as long as 16 slices, will averaging only instances of 16 slices enhance or decrease the performance? One may suspect that averaging over different lengths of instances will distort the data, though intuitively only averaging one length of instances may increase the error to detect the same syllable of other lengths.*

We share the reviewer’s intuition that restricting the sample size may have the effect of increasing the error. Also, we remind the reviewer that even single spectral slices are averages over some length of the acoustic signal, and that that the windowing inherent in calculating a non-overlapping short time spectrum is not precisely aligned with syllable onsets or offsets. However, to check, we reran our algorithm on a several syllables using only the slices from the modal length syllables as targets. As expected, there was no significant change in the performance of the algorithm.

*6. The offline success of this work may be compromised in the online process in actual experiments due to different asparagus, software, e.g., LabVIEW vs. MATLAB, and the requirement to deliver the triggered stimulus as soon as possible, i.e., balancing accuracy and efficiency. What will the detection performance be once the optimal template is applied in an experiment? Could it be the technical limitations after all to decide the hit rate and eventual delay in delivering the stimulus?*

We remind the reviewer that our algorithm is an offline algorithm whose sole purpose is to provide a better template to use in whatever online algorithm is being used within a real-time experiment. Any technicalities of real time implementation are shared by the original averaged template and the new optimized version. In general, simple online matching algorithms - such as the one we use - have a low computational cost and take less than 1-2 msec on a moderate speed computer. As for performance evaluations, our Matlab algorithm exactly mimics the popular real-time algorithm EvTAF, written in LabView. We have confirmed this match by running our triggering algorithm on data recorded during an EvTAF session.

*7. How long does it take to acquire an optimized template from raw recordings for one syllable? The authors may indicate this in the discussion as well.*

It generally takes less than 5 minutes to run the template optimization algorithm after the songs have been annotated. We have included this fact in our discussion.
